# How do oncology journals approach plagiarism? A website review

**DOI:** 10.1186/s41073-025-00160-4

**Published:** 2025-03-31

**Authors:** Johanna Goldberg, Heather Snijdewind, Céline Soudant, Kendra Godwin, Robin O’Hanlon

**Affiliations:** Memorial Sloan Kettering Cancer Center Library, 1275 York Ave, New York, NY 10065 USA

**Keywords:** Cancer research, Ethics, Plagiarise, Plagiarize, Plagiarism, Text similarity, Scholarly communications, Scientific misconduct, Research misconduct

## Abstract

**Background:**

Journals and publishers vary in the methods they use to detect plagiarism, when they implement these methods, and how they respond when plagiarism is suspected both before and after publication. This study aims to determine the policies and procedures of oncology journals for detecting and responding to suspected plagiarism in unpublished and published manuscripts.

**Methods:**

We reviewed the websites of each journal in the Oncology category of Journal Citation Reports' Science Citation Index Expanded (SCIE) to determine how they detect and respond to suspected plagiarism. We collected data from each journal's website, or publisher webpages directly linked from journal websites, to ascertain what information about plagiarism policies and procedures is publicly available.

**Results:**

There are 241 extant oncology journals included in SCIE, of which 224 (92.95%) have a plagiarism policy or mention plagiarism. Text similarity software or other plagiarism checking methods are mentioned by 207 of these (92.41%, and 85.89% of the 241 total journals examined). These text similarity checks occur most frequently at manuscript submission or initial editorial review. Journal or journal-linked publisher webpages frequently report following guidelines from the Committee on Publication Ethics (COPE) (135, 56.01%).

**Conclusions:**

Oncology journals report similar methods for identifying and responding to plagiarism, with some variation based on the breadth, location, and timing of plagiarism detection. Journal policies and procedures are often informed by guidance from professional organizations, like COPE.

**Supplementary Information:**

The online version contains supplementary material available at 10.1186/s41073-025-00160-4.

## Background

The Committee on Publication Ethics (COPE), an influential organization that includes more than 3,200 medical journals as members, defines plagiarism as "When somebody presents the work of others (data, words or theories) as if they were his/her own and without proper acknowledgment" [1, 2]. This definition, in line with those of other societies, such as All European Academies (ALLEA), leaves significant room for interpretation [3].

COPE's *Ethics Toolkit for a Successful Editorial Office* states, "What constitutes plagiarism and redundant/overlapping publication should be specified" by journals [4]. Specification is needed because the ways journals characterize plagiarism are not standardized, yet a 2021 study of 50 social sciences and 50 science journals found fewer than half of journals defined plagiarism in their author instructions, and just three detailed how the journal responds to plagiarism [5].

To respond to plagiarism, however defined, a journal must first identify it. The methods used by journals to detect plagiarism are varied, and may be manual, automated, or a combination of both. Automated detection tools—Crossref Similarity Check, iThenticate, and others—compare a manuscript to a large collection of articles, websites, and other published works. This comparison results in a similarity report, which provides a percentage of the manuscript that has been identified as similar to known texts, along with a delineation of which segments resemble published works [6]. While these tools are sometimes known as plagiarism detection software, they cannot always identify plagiarized ideas, articles that have been translated from another language without permission, stolen data, reproduced images and figures, or acceptable forms of paraphrasing [7–9].

The interpretation of a tool's similarity report and score is not standardized across journals, nor is the point in the editorial process when these tools are used. There are also differences in the procedures a journal follows once plagiarism is detected by the journal or reported by individuals. While many variables are at play—the timing of the discovery of plagiarism or other misconduct, notably fabrication or falsification—the policies of the journal are likely also a contributing factor. [10].

Plagiarism is a documented issue in oncology journals. At the time of writing, of the 3,014 articles in the field of oncology included in the Retraction Watch database, 283 (9.39%) were retracted due to plagiarism, with reasons for retraction coded as "duplication of text," "euphemisms for plagiarism," "plagiarism of article," or "plagiarism of text" [11]. When all reasons are considered, plagiarism-related and otherwise, oncology is the medical field with the most retractions [12, 13].

Given the discrepancies in defining, detecting, and responding to plagiarism, we undertook a study to determine how journals in oncology, our institution's focus, approach plagiarism.

## Methods

In August 2023, we searched Journal Citation Reports (JCR, Clarivate), the standard journal comparison and selection tool, for journals in the Oncology category of the Science Citation Index Expanded (SCIE). The JCR Year of 2022 was used, as it was the most recent year available.

In April 2024, we located the webpages where each journal on the JCR list provides information about their editorial policies and procedures around plagiarism or publication ethics. For the purposes of this study, "plagiarism policies" are defined as the set of rules or guidelines established by a journal to prevent and address plagiarism; "plagiarism procedures" are defined as the specific actions or steps a journal says it takes to detect plagiarism and to respond to it when it is detected; and these policies and procedures, as stated, must be present within or linked to from a journal's website. This means located webpages included journal-specific webpages within the journal website and publisher-level webpages directly linked from the journal website. We compiled these URLs in a Microsoft Excel spreadsheet for later data collection.

We built on this spreadsheet to develop a data collection instrument, which we pilot tested from May through June 2024, using a sample of 24 journals that reflected the variety of publishers in the full JCR journal list. We compared issues encountered during the pilot test as a group, followed by an analysis of data collection inter-rater variability. We adjusted the data collection instrument and wrote a guide to the data collection process in Microsoft Word (Appendix 1) to make the process as uniform as possible across reviewers.

In the data collection guide, we compiled a list of terms found on pilot test journal webpages or journal-linked publisher webpages that were iterations of the word plagiarism, plagiarism synonyms, or that commonly appeared within discussions of plagiarism. Plagiarism for our purposes is as defined by COPE, but with the addition of self-plagiarism and ethical issues tied to attribution. Self-plagiarism, also known as text recycling, is defined by COPE as "overlap of text with an author's own previously published work" [14]. "Ethical issues tied to attribution" refers to our interest in capturing sections of webpages that discuss publication ethics in the context of attribution, not publication ethics broadly, meaning describing ethical or unethical behavior and clearly including plagiarism. We defined a webpage mentioning plagiarism as including one or more terms from this list when located within the context of this expanded plagiarism definition or including the name or logo of a text similarity detection tool. The full list of terms is included in Appendix 1. We noted the tool names mentioned in writing or as software logos on these journal or journal-linked publisher webpages during data collection.

Website data collection occurred from June through July 2024. Data collection questions and selection options for reviewers are available in Appendix 1. Two reviewers independently collected data from each journal, which included updating webpage URLs where necessary. Conflicts within the quantitative data were resolved by consensus in August 2024. Three reviewers performed a qualitative analysis of collected data for the following: other methods used for checking for plagiarism; when in the process plagiarism detection is conducted; and possible outcomes when plagiarism is suspected or identified.

## Results

JCR includes 242 journals in the Oncology category of SCIE for 2022. We removed one that had ceased publication and included the remaining 241 in our study. A full list of these 241 journals is available in Appendix 2.

**Fig. 1:**
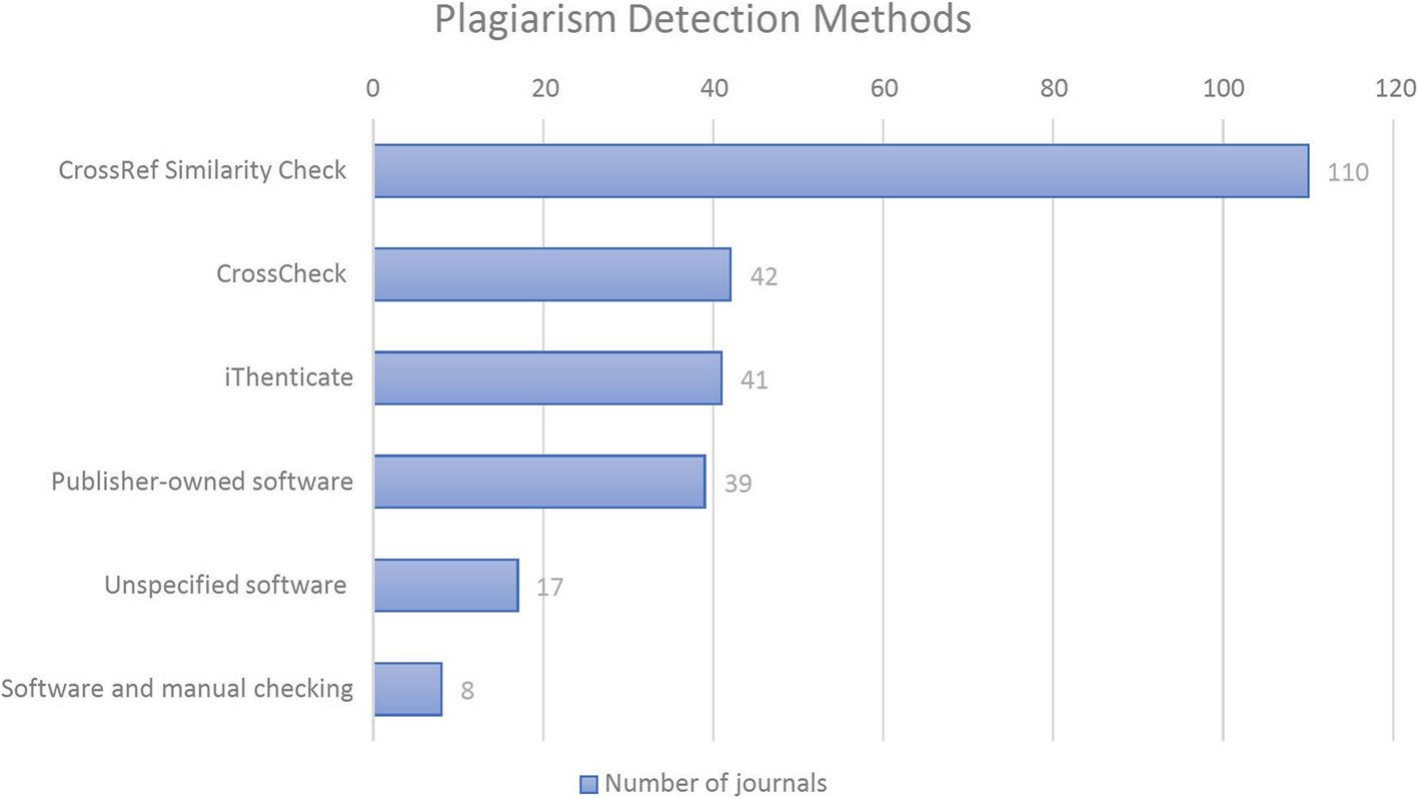
The plagiarism detection methods that journals indicate using, as stated on journal or journal-linked publisher webpages. Some journals list more than one method

The websites of 224 of the 241 journals (92.95%) mention plagiarism or directly link to publisher-level webpages that mention plagiarism. Of these 224 journals, 209 (93.3%, and 86.72% of the 241 total journals examined) mention checking submissions for plagiarism. Out of the 209 journals, 207 (99.04%, and 85.89% of the 241 total journals examined) give the method used for checking for plagiarism, with some listing more than one method. The most frequently mentioned method is Crossref Similarity Check (110 journals), followed by CrossCheck (42 journals), iThenticate (41 journals), a publisher-owned software (39 journals), and unspecified software (17 journals). Only one of the 224 journals (0.44%) details the number of plagiarized words or percentage of plagiarized text that would lead to further review for potential plagiarism. Eight journals of the 224 (3.57%) mention using both software and manual checking. Six of these further describe the manual checking process as utilizing search engines and PubMed, with two specifying performing title searches with these tools (Fig. 1: What Plagiarism Detection Methods Are Used?).

Of the 209 journals with websites or linked publisher webpages that mention checking submissions for plagiarism, 85 (40.67%, and 35.27% of the 241 total journals examined) provide some information about when in the editorial process the journal conducts its proactive plagiarism detection. Of these 85 journals, 21 (24.71%) explicitly state the stage at which plagiarism detection occurs, while 64 (75.29%) use less specific language from which we extrapolated when in the process it takes place. When a journal states it screens all submissions for plagiarism, we inferred that plagiarism screening happens at the manuscript submission or initial editorial review stages, before any manuscripts are rejected. [15, 16] When a journal states it screens all manuscripts submitted after peer review for plagiarism, we interpreted that it conducts plagiarism detection at or after revision submission.

**Fig. 2:**
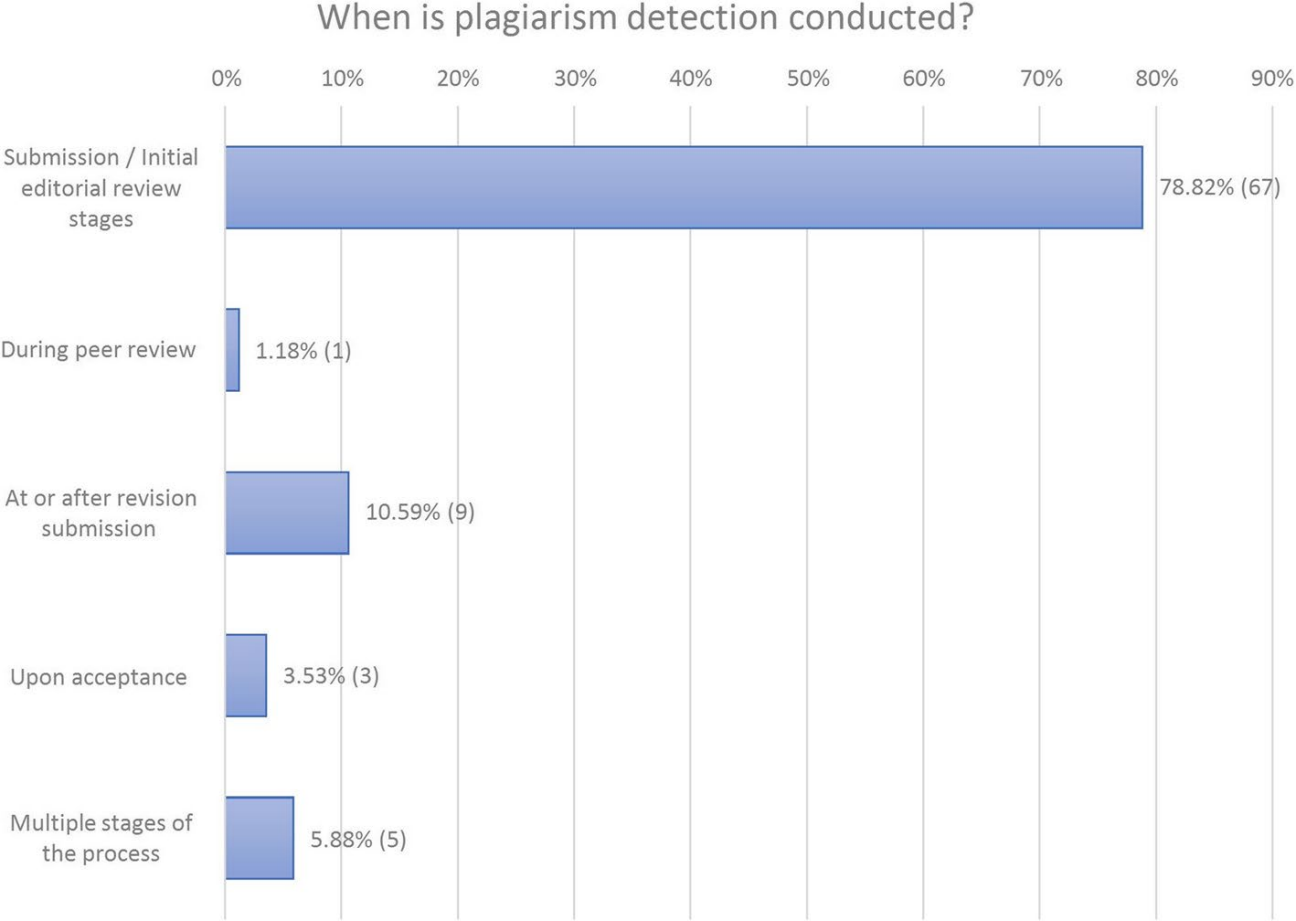
The timing of plagiarism detection that journals indicate using, as stated on journal or journal-linked publisher webpages. Some journals list more than one stage

Of the 85 journals that provide information about when they check for plagiarism, 67 (78.82%) indicate they conduct plagiarism detection at the manuscript submission or initial editorial review stages, one (1.18%) states it does so during the peer review process, 9 (10.59%) say they do so at or after revision submission, 3 (3.53%) indicate they do so upon acceptance of the manuscript, and 5 (5.88%) say they do so at multiple stages of the process (Fig. 2: When Is Plagisarm Detection Conducted?).

Possible outcomes when plagiarism is suspected or identified are outlined by 204 of the 224 journals whose websites or linked publisher webpages mention plagiarism (91.07%, and 84.65% of the 241 total journals examined), with some listing more than one outcome. These outcomes are grouped broadly, with some occurring at multiple points in the plagiarism detection process.

Journals within the 204 mention several steps they follow when plagiarism is suspected: contacting individuals outside of the journal (131, 64.21%), contacting authors (77, 37.74%), conducting or requesting investigations (76, 37.25%), rejecting or not considering a manuscript for publication (68, 33.33%), contacting individuals within the journal (19, 9.31%), and prohibiting future submissions to the journal and/or affiliated journals either within a given time period or indefinitely (10, 4.90%).

Journals within the 204 also list possible outcomes once plagiarism has been identified. For identified plagiarism in submitted manuscripts, these include the same listed outcomes as when plagiarism is suspected, but with the addition of posting additional types of corrections (e.g., clarification, apology, notice of misconduct, notice of withdrawal) (47, 23.04%) and penalizing or taking legal action against authors (14, 6.86%). For identified plagiarism in published articles, these include the same listed outcomes as submitted manuscripts, except for rejecting or not considering a manuscript for publication, and with the addition of retracting an article (128, 62.74%), posting errata or corrigenda (93, 45.59%), posting expressions of concern (68, 33.33%), following the policies of the journal (2, 0.98%), and removing further association with authors and institutions (1, 0.49%).

The most frequent possible outcome outlined within the 204 was the mention of following professional guidelines when plagiarism is suspected or identified, such as those from COPE (135, 66.18%), or another organization (e.g., Directory of Open Access Journals [DOAJ], International Committee of Medical Journal Editors [ICMJE], National Library of Medicine [NLM], Open Access Scholarly Publishing Association [OASPA], Office of Research Integrity [ORI], World Association of Medical Editors [WAME]) (34, 16.67%). Following these guidelines takes place when plagiarism is suspected or identified, both before and after publication.

## Discussion

As determined by our review of journal websites, 224 of the 241 oncology journals (92.95%) have a plagiarism policy or mention plagiarism; 207 of the 241 (85.89%) indicate using a text similarity software or other checking method.

The listing of multiple text similarity tools on journal or journal-linked publisher webpages shows a level of confusion with how these tools are labeled and named. CrossCheck, Crossref Similarity Check, and iThenticate are all products from the company Turnitin, with CrossCheck/Crossref Similarity Check marketed to publishers, iThenticate to authors, and Turnitin to academia [7]. CrossCheck changed its name to Crossref Similarity Check in 2016 [17]. The frequent naming of CrossCheck strongly suggests that webpages that discuss plagiarism are not frequently reviewed or updated. It is unclear if this is an oversight, or if it indicates a more serious issue with the journals' commitments to preventing plagiarism.

The published literature has numerous examples of journals determining how to best use text similarity software and what software-determined similarity scores should be a cause for further examination [18–20]. There are also many calls for editorial assessment of the automated process, especially urges to not reject manuscripts without first reviewing the report [8, 9, 21, 22]. The near absence of similarity score information on journal or journal-linked publisher webpages could be indicative of these concerns. Journals may not want to publicize the similarity report percentages that lead to additional scrutiny to prevent authors from circumventing them. Alternately, it may indicate that these reports are not used as the sole method for determining the presence or plagiarism, or that journals have leeway when it comes to their implementation.

Clearly, context matters, as does extent. Some have argued that only intentional plagiarism should count as misconduct, as unintentional copying is "ubiquitous." [18] Others have said that reusing some text without proper citation, which can be detected by software, is less egregious than copying ideas without citation, which cannot be [19]. While technology may reveal both intentional and unintentional text duplication, it does not address the root causes of plagiarism, like the pressure to publish (in English, no matter an author's native language) and power structures that allow for "intellectual exploitation," like stolen ideas [20]. COPE's "Text recycling guidelines for editors" key points state, "it may be entirely appropriate to have overlap in a methods section of a research article (referring to a previously used method) with citation of the original article." The full document leaves the amount of acceptable self-plagiarism to the judgement of the editors while continuing to stress the importance of citation [15]. What is and is not considered plagiarism may vary from person to person, journal to journal, and culture to culture, confounding the issue [21, 22].

### Limitations

This study only assessed plagiarism policies and procedures in one discipline; its generalizability to other areas is unknown. We used journal website navigation menus and clearly labeled hyperlinks to identify webpages where journals and publishers provide information about their plagiarism policies and procedures; we did not exhaustively search journal or publisher websites for this information. Websites change frequently, and our study was only able to capture one snapshot in time. A website can only say so much about a journal; our analysis is limited to publicly available information on journal and journal-linked publisher webpages that may diverge from actual journal practices and resulting outcomes in response to plagiarism. We did not compare the listed journal policies and procedures to journal responses to past cases of plagiarism, nor did we capture data specific to AI-related plagiarism, a growing issue that is currently difficult to detect [23, 24].

## Conclusion

Our study found similarities in the ways oncology journals say they detect and respond to suspected plagiarism. Responses may be impacted by context—where in the manuscript plagiarism occurs, when in the publication process the suspected plagiarism was detected—and are often guided by the standards of professional publishing organizations. Further research could determine whether or how generative AI is changing the editorial process; explore beyond a single medical field; and compare stated policies with responses to known instances of plagiarism.

## Supplementary Information

Supplementary Material 1.

Supplementary Material 2.

## Data Availability

The dataset analyzed during the current study is available from the corresponding author on reasonable request.

## Abbreviations

ALLEA: All European Academies
COPE: Committee on Publication Ethics
DOAJ: Directory of Open Access Journals
ICMJE: International Committee of Medical Journal Editors
JCR: Journal Citation Reports
NLM: National Library of Medicine
OASPA: Open Access Scholarly Publishing Association
ORI: Office of Research Integrity
SCIE: Science Citation Index Expanded
WAME: World Association of Medical Editors
