# Supplementary material for: How do oncology journals approach plagiarism? A website review
Source: Res Integr Peer Rev. 2025 Mar 31;10:3. doi: 10.1186/s41073-025-00160-4 (PMC11956406; doi:10.1186/s41073-025-00160-4)
Supplement: Supplementary file 1 — Supplementary Material 1. [file 41073_2025_160_MOESM1_ESM.docx]

**How do Oncology Journals Approach Plagiarism? A Website Review**

**Appendix 1: Guide to the Journal Website Data Collection Process**

| **Data Collection Questions** | **Data Collection Selection Options** | **Data Collection Guidelines for Reviewers** |
| --- | --- | --- |
| URLs of webpages within journal and/or publisher websites where information about editorial policies and procedures around plagiarism or publication ethics was found by reviewers | URL | While each of the data collection questions refers to a singular webpage, please check all available URLs for each question, and document where something was found within the corresponding notes field for the question if you think this would be helpful for reviewers during the data conflict resolution stage.  If any of the URLs do not work, or if you find information on a URL not listed, update accordingly within the data collection instrument. Context and instructions below.   - During the original URL collection, URLs for publisher-level webpages were added when the journal-specific website linked directly to them/directed authors who might be seeking info on plagiarism policies there. - In cases where there is only a URL for a publisher-level webpage for a given journal, double-check that the journal-specific website links directly to it. - If you come across a journal-specific website that links to a publisher-level webpage for their complete plagiarism/redundant publication policy, but this URL for the publisher-level webpage is missing, add this URL and note what was done within General Notes. |
| "Does the webpage mention plagiarism?" | Yes No | If plagiarism/similarity checking software is mentioned in general or by name, even without any additional mention of plagiarism, select Yes.  If the webpage includes a plagiarism detection software logo, even without mentioning the software in the text (often Springer), select Yes.  If the webpage says the journal/publisher (often BMC) is a member of "CrossCheck's plagiarism detection initiative," select Yes.  Search for words like plagiari*, similar*, screen*, check*, pass off, duplicat*, misconduct*, original*, attribut*, integrity, recycl*, redundan*, reproduc*, copy, copie*, ethic*, unethic*, dual. If found, review the context to confirm whether the webpage uses the term in reference to plagiarism.   - If ethics or misconduct are only mentioned generally without clearly including plagiarism, select No. - If 'duplicate' is used to refer to submitting to multiple journals at one time, select No. - If 'duplicate' is used to refer to including text that has been previously published, select Yes. - If text recycling/self-plagiarism is mentioned, select Yes. |
| "Does the webpage mention checking submissions for plagiarism?" | Yes  No | If a software or any other form of checking is mentioned, even if not every submission is checked, select Yes. |
| "Does the webpage give the method(s) used for checking for plagiarism?" | Yes-CrossCheck Yes-Crossref Similarity Check Yes-DupliChecker Yes-Grammarly  Yes-iThenticate Yes-Manual checking  Yes-Other Yes-Spot checking a selection of submissions Yes-Turnitin  Yes-Unspecified software No | If the webpage says "Crossref Similarity Check Powered by iThenticate," select Yes-Crossref Similarity Check.  If the webpage says only 'iThenticate,' select Yes-iThenticate.  If the webpage says the journal/publisher is a member of "CrossCheck's plagiarism detection initiative" (often BMC), select Yes-CrossCheck. If it also links to Crossref Similarity Check, add that to the notes field.  If the webpage includes a logo of a plagiarism detection software even without mentioning it in the text (often Springer), select that software from the list of options.  If the webpage says "this journal uses iThenticate's CrossCheck software" (often Wiley), select Yes-CrossCheck.  If not all submissions are checked/submissions 'may' be checked, select the method stated from the list of options, and add the additional information in the notes field.  If a software and manual review is specifically mentioned, select Yes-Other, and enter the details in the notes field. |
| Notes on "Does the webpage give the method(s) used for checking for plagiarism?" | Free text | See guidelines for "Does the webpage give the method(s) used for checking for plagiarism?" |
| "Does the webpage say when in the process plagiarism detection is conducted?" | Yes No | If Yes is selected, the following notes field is required.  This question refers to the point when the journal (pro)actively checks or screens for plagiarism.   - If the webpage says "manuscripts are/may be checked" without further details, select No. - If the webpage says "all manuscripts/submissions" are checked/screened, select Yes. This language strongly implies that screening occurs early in the journal's review process, most likely at submission or initial editorial review (and before peer review). - If the webpage says manuscripts are checked/screened "after peer review," select Yes. This language implies that screening occurs late in the process, most likely at revision submission or acceptance.     For reference, general flowcharts of typical journal review processes can be found [here](https://authorservices.wiley.com/Reviewers/journal-reviewers/what-is-peer-review/the-peer-review-process.html) and [here](https://www.biomedcentral.com/getpublished/peer-review-process#:~:text=How%20peer%20review%20works) (the exact process will vary from journal to journal). (the exact process will vary from journal to journal). |
| Notes on "Does the webpage say when in the process plagiarism detection is conducted?" | Free text | Enter the text from the webpage that indicates when detection is conducted. |
| "Does the webpage detail the number of plagiarized words or percentage of plagiarized text that would lead to further review for potential plagiarism?" | Yes  No | If Yes is selected, the following notes field is required. |
| Notes on "Does the webpage detail the number of plagiarized words or percentage of plagiarized text that would lead to further review for potential plagiarism?" | Free text | Enter the specific number or percentage from the webpage. |
| "Does the webpage outline possible outcomes when plagiarism is suspected or identified?" | Yes  No | If the website mentions following the Committee on Publication Ethics (COPE) in cases of suspected or identified plagiarism in a manuscript or a published article, select Yes.  If possible outcomes are listed as things that may happen, select Yes. |
| Notes on "Does the webpage outline possible outcomes when plagiarism is suspected or identified?" | Free text | Cut/paste the text from the webpage that gives the possible outcomes.  If text does not mention outcomes but is otherwise of interest, feel free to include it here. |
| General Notes | Free text | If you are unsure/have questions about your responses to any of the fields, add a note here. Also add a note if any information strikes you as worth mentioning that does not fit in the other fields. |
